# Microbes on the Cliff: Alpine Cushion Plants Structure Bacterial and Fungal Communities

**DOI:** 10.3389/fmicb.2013.00064

**Published:** 2013-03-27

**Authors:** J. Roy, C. H. Albert, S. Ibanez, P. Saccone, L. Zinger, P. Choler, J.-C. Clément, S. Lavergne, R. A. Geremia

**Affiliations:** ^1^UMR CNRS-UJF 5553, Laboratoire d’Ecologie Alpine, Université de GrenobleGrenoble, France; ^2^UMS CNRS-UJF 3370, Station Alpine J. Fourier, Université de GrenobleGrenoble, France

**Keywords:** soil microbial communities, beta-diversity, elevation gradients, ecosystem engineering, foundation species, molecular fingerprint, alpine ecosystems, *Silene acaulis*

## Abstract

Plants affect the spatial distribution of soil microorganisms, but the influence of the local abiotic context is poorly documented. We investigated the effect of a single plant species, the cushion plant *Silene acaulis*, on habitat conditions, and microbial community. We collected soil from inside (In) and outside (Out) of the cushions on calcareous and siliceous cliffs in the French Alps along an elevation gradient (2,000–3,000 masl). The composition of the microbial communities was assessed by Capillary-Electrophoresis Single Strand Conformation Polymorphism (CE-SSCP). Univariate and multivariate analyses were conducted to characterize the response of the microbial beta-diversity to soil parameters (total C, total N, soil water content, $\text{N}-{\text{NH}}_{4}^{+},\;\text{N}-{\text{NO}}_{3}^{-}$, and pH). Cushions affected the microbial communities, modifying soil properties. The fungal and bacterial communities did not respond to the same abiotic factors. Outside the cushions, the bacterial communities were strongly influenced by bedrock. Inside the cushions, the bacterial communities from both types of bedrock were highly similar, due to the smaller pH differences than in open areas. By contrast, the fungal communities were equally variable inside and outside of the cushions. Outside the cushions, the fungal communities responded weakly to soil pH. Inside the cushions, the fungal communities varied strongly with bedrock and elevation as well as increases in soil nutrients and water content. Furthermore, the dissimilarities in the microbial communities between the In and Out habitats increased with increasing habitat modification and environmental stress. Our results indicate that cushions act as a selective force that counteracts the influence of the bedrock and the resource limitations on the bacterial and fungal communities by buffering soil pH and enhancing soil nutrients. Cushion plants structure microbial communities, and this effect increases in stressful, acidic and nutrient-limited environments.

## Introduction

Soil microbial communities are a major component of the biosphere and play a critical role in nutrient cycling and ecosystem functioning (van der Heijden et al., 2008). Consequently, identifying the key factors that control their composition is of great interest. The recent development of molecular tools has permitted a more thorough study of these communities. Abiotic factors such as soil pH and soil nutrient availability have been identified as key determinants of the richness and composition of microbial communities (Fierer and Jackson, 2006; Lauber et al., 2008). The changes in these factors along elevation gradients have been shown to drive compositional changes in microbial communities (Bryant et al., 2008; Fierer et al., 2011; Singh et al., 2012; Wang et al., 2012). In addition, single plant affects the composition of soil microbial communities (Kowalchuk et al., 2002) and plant cover is known to be a central driver of the spatial distribution of soil microorganisms (Eskelinen et al., 2009; Zinger et al., 2011). This status could result from the direct effect of species-specific mutualistic associations (Berg and Smalla, 2009; Hartmann et al., 2009) or from an indirect effect, as plants influence the physico-chemical properties of soil through litter deposition and root exudation of organic compounds (Eviner and Chapin, 2003; Bais et al., 2006). Despite recent insights into the biotic and abiotic factors affecting microbial communities (Zinger et al., 2011), three main issues impede our understanding of the underlying processes: (i) the complexity of the systems that are typically surveyed, which often involve multiple plant species and mature soils; (ii) the strong connection between soils and plant characteristics; and (iii) the lack of comparative analyses along environmental gradients that would allow us to assess how ecological processes can be affected by changes in abiotic variables.

We propose that cushion plants are an ideal model to simultaneously address these limitations and better understand how the interactions between biotic and abiotic factors may structure soil microbial communities. Cushion plants are a common growth form in alpine ecosystems (Körner, 2003) and provide a natural system with three main characteristics: (i) they are single or very dominant plants in a highly mineral matrix and can be seen as unique fertility hotspots in the desert, particularly in rocky landscapes with alpine cliffs where plant cover is very sparse or non-existent (Körner, 2003); (ii) they present a *de novo* soil formation resulting solely from the accumulation of its own living and dead tissues, thus reducing the confounding effect of other carbon sources that occur in mature soils; and (iii) they are broadly distributed along strong environmental gradients (e.g., elevation, bedrock). High mountain environments are characterized by low air and soil temperatures, high levels of solar radiation and wind exposure and strong effects on biotic communities (Körner, 2003). Consequently, cushion plants can be studied across broad altitudinal gradients to better understand how temperature and other associated abiotic factors that change with elevation can influence community and ecosystem properties. For these reasons, cushion plants have been extensively studied to determine how a single organism may modify local habitat conditions with consequences for the distribution and performance of other organisms. This type of non-trophic ecological interaction that strongly affects the community structure is referred to as ecosystem engineering (Jones et al., 1994, 1997). The low, compact stature and thick canopy of cushion plants is known to buffer temperatures and increase nutrient availability and water content compared to adjacent open areas (Arroyo et al., 2003; Cavieres et al., 2007; Yang et al., 2010; Anthelme et al., 2012). They are known to positively affect the richness of local plant (Badano et al., 2002; Arroyo et al., 2003; Badano and Cavieres, 2006; Cavieres et al., 2006; Antonsson et al., 2009; Sklenar, 2009; Yang et al., 2010; Anthelme et al., 2012; Molenda et al., 2012) and arthropod communities (Molina-Montenegro et al., 2006; Molenda et al., 2012). For instance, by maintaining their effect on temperature and nutrients, the positive effect of cushions on neighboring plants is accentuated by environmental stress (Arroyo et al., 2003; Badano and Cavieres, 2006; Antonsson et al., 2009; Yang et al., 2010; Anthelme et al., 2012). One can assume that cushion plants should similarly structure the composition of microbial communities within cliff soils along environmental gradients. Inside the cushion, we predict a convergence of microbial communities due to the local environmental buffering that is created by the cushion plant. Outside the cushion, we anticipate contrasting communities due to their response to bedrock type and elevation.

The goal of this study was to determine the extent to which cushions affect the abiotic characteristics of the surrounding soils and the associated bacterial and fungal communities along the elevation gradient and on different bedrock types. We chose *Silene acaulis* (Caryophyllaceae) as our study species. This alpine species is common in fell-fields and cliff ecosystems. It forms large cushions (up to 60 cm in diameter), occurs over a large elevation range (from nearly 2000 to 3000 masl) and is able to grow on both calcareous and siliceous bedrocks. We collected soil from inside and outside of *S. acaulis* cushions on highly rocky slopes and cliffs, along replicated elevational transects in two mountains ranges that differed in bedrock type.

## Materials and Methods

### Study site and sample collection

**Figure 1:**
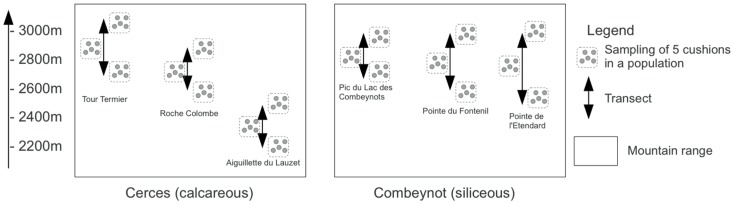
**Sampling design of the study**. Replicated elevational transects are named CI, CII, and CIII for the Tour Termier, Roche Colombe, and Aiguillette du Lauzet, respectively in the calcareous Cerces Mountains, and SI, SII, and SIII for Pic du Lac des Combeynots, Pointe du Fontenil, and Pointe de l’Etendard, respectively in the siliceous Combeynots Mountains.

The fieldwork was conducted near the Lautaret Pass and the Station Alpine Joseph Fourier in the southern French Alps (Hautes-Alpes, 05) during September 2009. We investigated patches of cushion plants distributed along elevation gradients on steep, south-facing, rocky slopes and cliffs ranging from 2,000 to 3,000 masl. The sampling was conducted in two distinct mountain ranges: the calcareous Cerces and the siliceous Combeynots Mountains (Figure 1). In each mountain range, we selected three summits for sampling along elevation transects (summit names are indicated in Figure 1 and are referred to as CI, CII, CIII and SI, SII, SIII for calcareous and siliceous massifs, respectively). On each summit, we sampled three populations of *S. acaulis* along elevation (Figure 1). For each population, five cushions were randomly selected. For each cushion, three soil cores were sampled inside the cushion (In habitat), and three cores were collected 10–20 cm away from the cushion’s edge (Out habitat). The three soil cores were pooled to yield five composite samples per habitat type and population. Soil cores were 5 cm deep and 5 cm in diameter. Due to the highly constrained sampling conditions, soil cores intended for microbial DNA analysis were conditioned in silica gel.

### Characterization of abiotic conditions

Fresh soil sub-samples were used for measurements of the gravimetric soil water content (SWC) at 105°C and the soil pH in the water (Robertson et al., 1999). The total C and N soil contents were measured with a FlashEA 1112 CN elemental analyzer (Thermo Fisher Scientific, Waltham, MA, USA). Fresh soil sub-samples were also extracted for ${\text{NO}}_{3}^{-}$ and ${\text{NH}}_{4}^{+}$ analysis by shaking for 1 h in 2 M KCl at 20°C, followed by filtration through Whatman paper. Soil extracts were analyzed for ${\text{N - NO}}_{3}^{-}$ and ${\text{N - NH}}_{4}^{+}$ using standardized protocols on a Flow Solution IV colorimetric chain (OI-Analytical Corp., College Station, TX, USA).

### Microbial community analysis

The molecular profiles of bacterial and fungal communities were obtained by Capillary-Electrophoresis Single Strand Conformation Polymorphism (CE-SSCP), a method that does not permit the identification of microbial taxa but instead provides a high resolution, reproducible picture of microbial communities for a large number of samples (Zinger et al., 2007, 2008, 2011). Soil DNA extractions were completed with the PowerSoil Well Soil DNA Isolation Kit (MO BIO Laboratoires, Ozyme, St. Quentin en Yvelines, France). Because the DNA extraction kit was designed for wet soils, we performed extractions with varying masses of dry soil, followed by DNA quantification with a NanoDrop ND 1000 (NanoDrop Technologies) and immediate verification by 1% agarose gel electrophoresis. Successful extraction was obtained with 0.07–0.10 g of soil. We amplified the V3 region of bacterial 16S RNA genes with the primers W49 (5′-ACGGTCCAGA CTCCTACGGG-3′) and W104-FAM labeled (5′-TTACCG CGGCTGCTGGCAC-3′) (Delbes et al., 2000) and the fungal ITS1 (Internal Transcribed Spacer) region with the primers ITS5 (5′ GGAAGTAAAAGTCGTAACAACG-3′) and ITS2-HEX labeled (5′-GCTGCGTTCTTCATCGATGC-3′) (White et al., 1990). The PCR reactions (25 μl) contained 2.5 mM MgCl_2_, 1 U AmpliTaq GoldTM buffer, 20 g l^−1^ bovine serum albumin, 0.1 mM each dNTP, 0.26 mM each primer, 2 U AmpliTaq Gold DNA polymerase (Applied Biosystems, Courtaboeuf, France) and 10 ng DNA template. PCR was performed as follows for bacteria: an initial step at 95°C (10 min), followed by 30 cycles at 95°C (30 s), 56°C (15 s), and 72°C (20 s) and a final step at 72°C (7 min). For fungi, the PCR protocol was as follows: an initial step at 95°C (10 min), followed by 33 cycles at 95°C (30 s), 54°C (15 s), and 72°C (30 s) and a final step at 72°C (7 min). Community molecular fingerprints were obtained by submitting the PCR products to CE-SSCP analysis as previously described (Zinger et al., 2007). The fluorescence profiles corresponded to the abundance of sorted DNA fragments according to their length and nucleotide composition.

Capillary-Electrophoresis Single Strand Conformation Polymorphism analysis was performed on an ABI Prism 3130 XL genetic analyzer (Applied Biosystems, Courtaboeuf, France) as described elsewhere (Zinger et al., 2008) and were visually checked individually. CE-SSCP profiles were normalized prior to statistical analysis.

### Data analysis

The abiotic characteristics were analyzed according to a generalized linear mixed model (Pinheiro and Bates, 2000) to account for the hierarchical sampling design and to test for the effect of the habitat type (In vs. Out), bedrock type (calcareous vs. siliceous), elevation (continuous, from approximately 2000 to 3000 m) and their interactions (“lmer” function of the “lme4” R package). Population (nested in Transect) and Transect (nested in Massif) levels were included as random factors, and elevation was defined as varying between transects. We tested the null model with random effects only and models that included all of the interactions terms for the fixed effects. The normality of the random factor and residuals were checked. The best models were selected according to AIC criteria (Akaike, 1974). The models were fitted using a maximum likelihood analysis for the comparison of nested models (Bates, 2010).

Pair-wise dissimilarities between microbial SSCP profiles were calculated with the Bray–Curtis distance (Legendre and Legendre, 1998). The resulting dissimilarity matrices were ordinated by Principal Coordinate Analysis (PCoA) (Legendre and Legendre, 1998). We used a vector-fitting approach to identify the directions in the microbial ordination space toward which a given environmental variable changed the most (Oksanen et al., 2011). To test the respective effects of habitat, elevation, and bedrock and their interactions on the microbial assemblage variation, we performed a non-parametric multivariate analysis of variance (Anderson, 2001) using the “adonis” function in the “vegan” R package (Oksanen et al., 2011). To test whether the significance of the factors changed with the spatial scale, we permutated samples between all conditions (within Population, within Transect, and within Massif) using the “strata” argument of the “adonis” function when appropriate. The non-parametric multivariate analysis was designed to test for differences in groups’ centroids, that is, a strict difference in community composition. However, the test is sensitive to differences in the multivariate dispersion from the centroid (Anderson, 2001). To disentangle both phenomena and because differences in multivariate dispersion are important for understanding the ecological effect of a factor, we tested whether the multivariate dispersion value differed between In and Out habitats (Anderson et al., 2006) using the “betadisper” function of the “vegan” R package. Multivariate dispersion is a measure of beta-diversity (Anderson et al., 2006). Mantel tests were used to assess the correlation between the dissimilarities and environmental distances of the microbial communities (Legendre and Legendre, 1998). All factors were tested for significance using the mean of 999 Monte-Carlo permutations. Finally, we measured the Bray–Curtis distance between the In and Out communities for each individual cushion according to the sampling design (hereafter, In-Out beta-diversity). We analyzed the relationship between bacterial and fungal In-Out beta-diversity and environmental dissimilarities as well as bedrock (considered as a factor with two levels) and elevation (considered as a continuous variable) using generalized linear mixed models, as described above, for the abiotic parameters.

All of the statistical analyses were conducted with R 2.13.0 software (R Development Core Team, 2011) using the packages “lme4” 0.999999-0 (Bates et al., 2011), “stats” (R Development Core Team, 2011), and “vegan” 1.17-11 (Oksanen et al., 2011).

## Results

### Soil characteristics and environmental context

**Figure 2:**
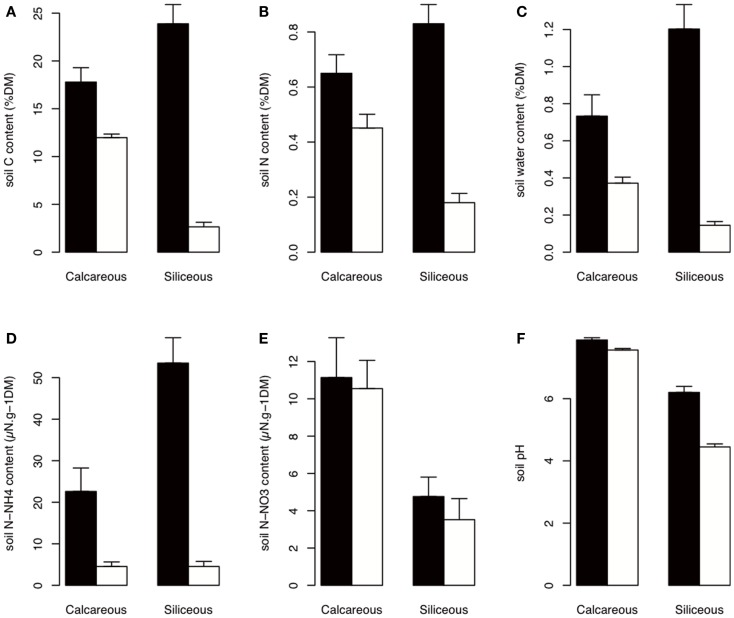
**Soil properties according to habitat and bedrock type**. The best AIC models indicated a habitat effect that is dependent on bedrock type (Table A1 in Appendix). Black: In habitat; white: Out habitat. Error bars represent SD. **(A–F)** are total carbon content, total nitrogen content, soil water content, ammonium content, nitrate content, and soil pH, respectively. DM, dry matter.

**Figure 3:**
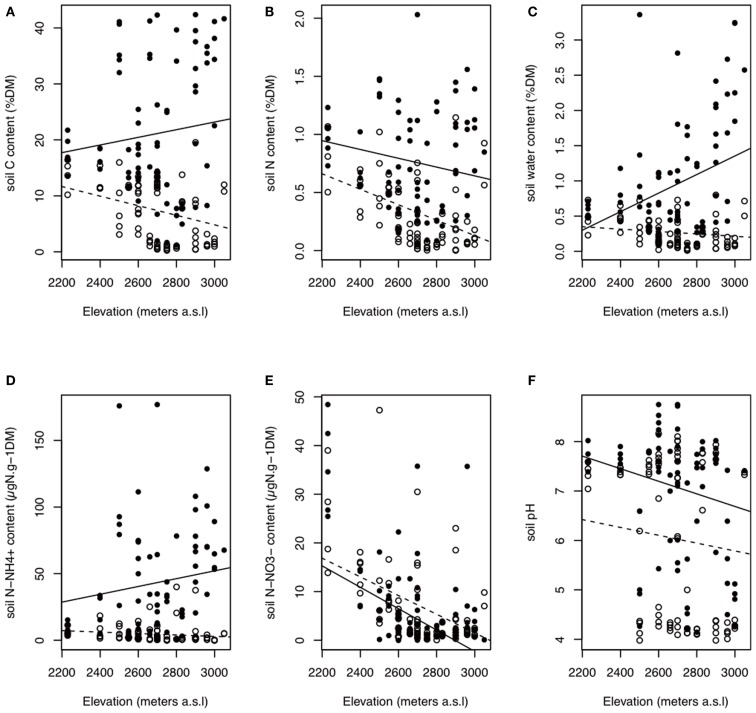
**Variations in soil properties according to habitat type and elevation**. The best AIC models indicated a habitat effect that is dependent on elevation. Black points with filled line: in habitat; white points with dashed line: out habitat. **(A–F)** are total carbon content, total nitrogen content, soil water content, ammonium content, nitrate content, and soil pH, respectively. Elevation did not affect the soil pH (Table A1 in Appendix). The observed trend between soil pH and elevation could come from the confounding effect of bedrock and elevation on pH because siliceous samples are, on average, higher in elevation. DM, dry matter.

The soil nutrients were influenced by bedrock type, habitat, and elevation (Figures 2 and 3; Table A1 in Appendix). The C, N, H_2_O, and ${\text{N - NH}}_{4}^{+}$ contents of In habitat were (i) higher than those of Out habitat, (ii) higher on siliceous bedrock than on calcareous bedrock and (iii) increased with elevation (Figures 2 and 3; Table A1 in Appendix). Conversely, the C, N, and H_2_O contents of Out habitat were (i) higher on calcareous than on siliceous bedrock (Figure 2) and (ii) decreased with increasing elevation (Figure 3; Table A1 in Appendix). It should be noted that in samples from the calcareous bedrock the determination of total carbon likely includes significant inorganic carbonates; although the difference in total carbon between the In and Out samples reflects primarily an increase in organic carbon for the In samples. Soil ${\text{N - NO}}_{3}^{-}$ was highly variable (Table A1 in Appendix). Soil ${\text{N - NO}}_{3}^{-}$ was mainly higher on calcareous bedrock and decreased with elevation (Figures 2 and 3; Table A1 in Appendix). The soil pH was strongly determined by bedrock type and was higher on calcareous bedrock. The measured differences in Out habitats between both bedrocks were strongly buffered inside cushions, with pH strongly increasing in In habitat of siliceous bedrock (Figure 2). Soil pH was not affected by elevation (Table A1 in Appendix).

### Bacterial patterns

**Figure 4:**
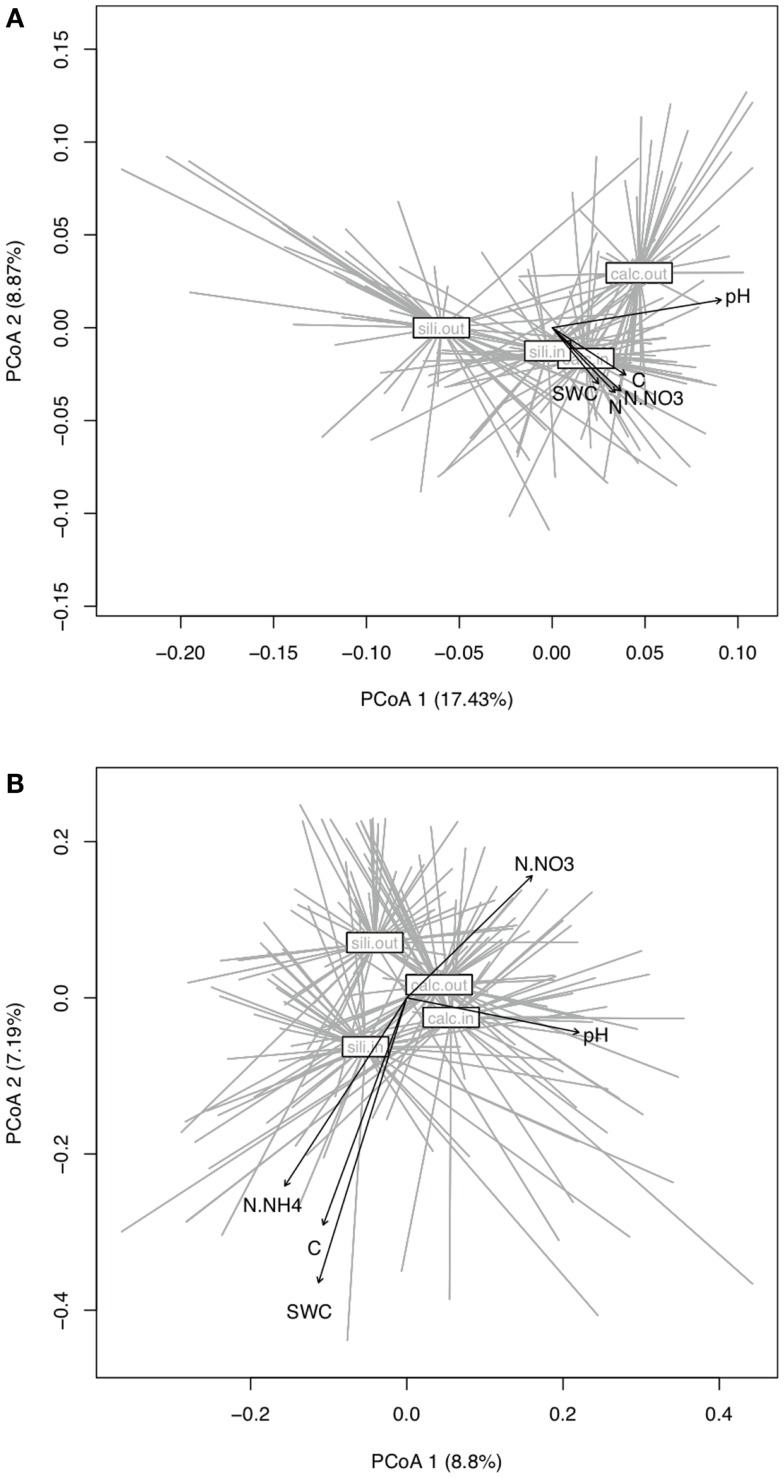
**Principal Coordinate Analysis (PCoA) of the bacterial (A) and fungal (B) dissimilarity matrices (Bray**–**Curtis) and vector-fitting of the environmental variables**. Communities were grouped to the centroid by bedrock and habitat. Arrows represent environmental variables that correlate significantly with the sample coordinates (*P* < 0.001, 999 Monte-Carlo permutations).

**Figure 5:**
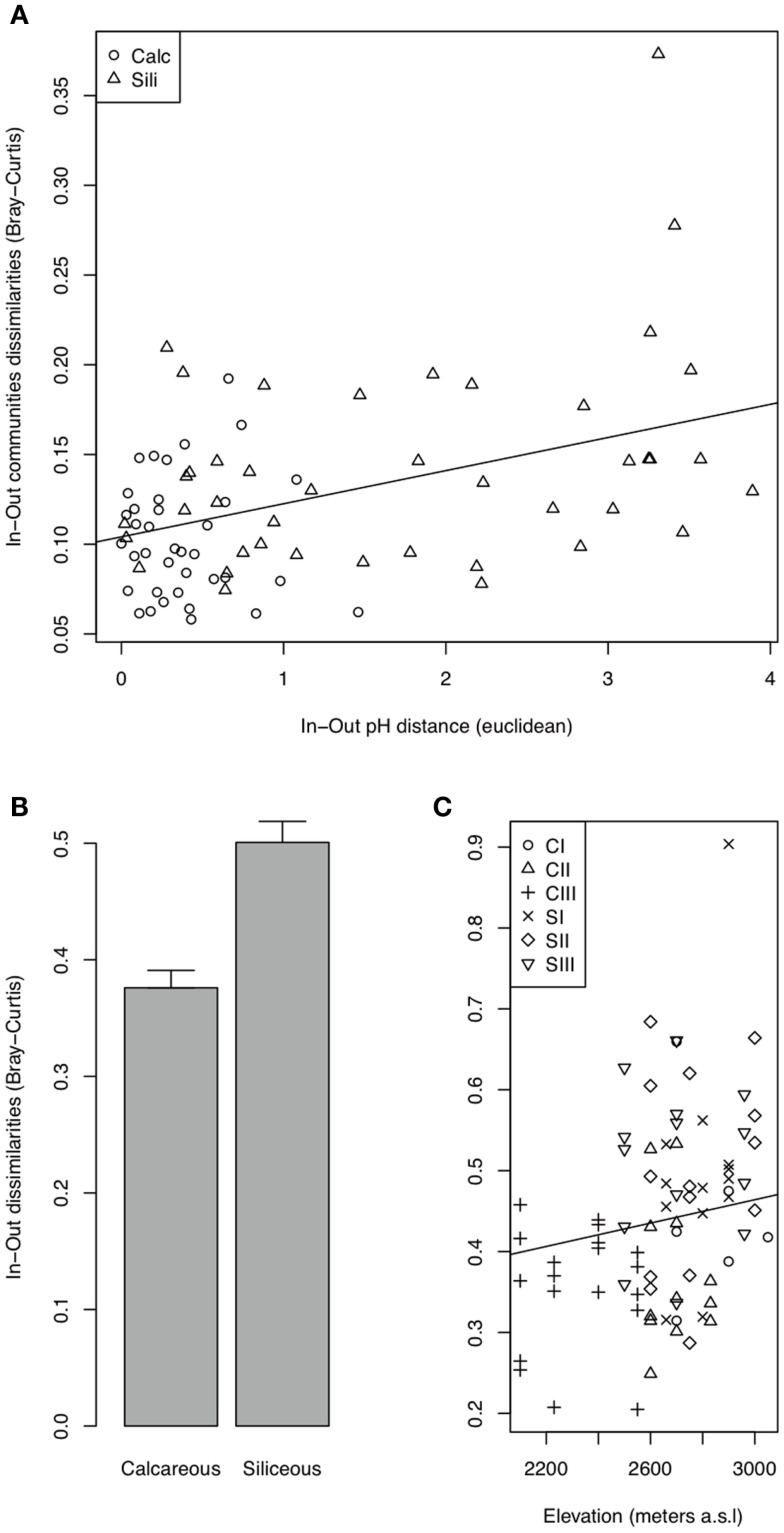
**Bacterial (A) and fungal (B,C) In-Out beta-diversity**. The best AIC models indicated a relationship between the In-Out beta-diversity and the In-Out pH distance (Euclidean) for bacteria and the effect of bedrock and elevation, without an interaction for fungi (Table A2 in Appendix).

The bacterial communities were mainly and significantly affected by bedrock types (*F*-ratio = 18.262, *R*^2^ = 0.088, *P* = 0.001; Table 1), as illustrated in the PCoA ordination and environmental fitting, which highlights the great dissimilarities between siliceous and calcareous Out communities along the pH gradient (*R*^2^ = 0.27, *P* = 0.001). Variation partitioning revealed a significant effect of habitat on bacterial community dissimilarity variation (*F*-ratio = 5.902, *R*^2^ = 0.028, *P* = 0.001; Table 1), regardless of the strategy that was adopted in the permutation procedure (Table A3 in Appendix). Calcareous and siliceous In communities were more similar and less variable than Out communities (avg. distance to centroid: Out = 0.09782, In: 0.07812, *F*-value = 17.1, *P* < 0.001; Figure 4A). Communities in In habitats displayed intermediate features along the pH gradient on the PCoA ordination and differentiated from Out communities along soil nutrient and water content gradients (${\text{N - NO}}_{3}^{-}$: *R*^2^ = 0.08, *P* = 0.002; N: *R*^2^ = 0.074, *P* = 0.004; C: *R*^2^ = 0.07, *P* = 0.006; SWC: *R*^2^ = 0.05, *P* = 0.02; Figure 4A). Furthermore, the In-Out beta-diversity was related to the paired In-Out pH distance and was higher and more pronounced on siliceous bedrock (Figure 5A; Table A2 in Appendix). Finally, the bacterial communities were marginally affected by elevation, and this response was dependent on the bedrock type (Table 1). A detailed analysis using the Mantel test revealed that bacterial communities respond to elevation solely in Out habitat on siliceous bedrock (Out habitat: Spearman rank ρ = 0.12, *P* = 0.03 and ρ = 0.05, *P* = 0.22 for siliceous and calcareous bedrock, respectively; In habitat: *P* > 0.05 for siliceous and calcareous bedrock).

**Table 1:** **Respective effects of habitat, bedrock, and elevation on microbial beta-diversity, as assessed by variation partitioning on Bray–Curtis dissimilarity matrices obtained from CE-SSCP profiles**.

Taxa	Factors	Df	SS	MS	FR	*R*^2^	Pr(>*F*)
Bacteria	Habitat	1	0.046	0.046	5.902	0.028	0.001
	Bedrock	1	0.142	0.142	18.262	0.088	0.001
	Elevation	1	0.026	0.026	3.302	0.016	0.004
	Habitat: bedrock	1	0.068	0.068	8.747	0.042	0.001
	Habitat: elevation	1	0.005	0.005	0.697	0.003	0.666
	Bedrock: elevation	1	0.015	0.015	1.876	0.009	0.070
	Habitat: bedrock: elevation	1	0.012	0.012	1.563	0.008	0.126
	Residuals	168	1.304	0.008	NA	0.806	NA
	Total	175	1.617	NA	NA	1.000	NA
Fungi	Habitat	1	0.552	0.552	5.004	0.026	0.001
	Bedrock	1	0.644	0.644	5.843	0.030	0.001
	Elevation	1	0.663	0.663	6.014	0.031	0.001
	Habitat: bedrock	1	0.259	0.259	2.353	0.012	0.002
	Habitat: elevation	1	0.137	0.137	1.244	0.006	0.199
	Bedrock: elevation	1	0.232	0.232	2.102	0.011	0.010
	Habitat: bedrock: elevation	1	0.182	0.182	1.654	0.009	0.041
	Residuals	168	18.522	0.110	NA	0.874	NA
	Total	175	21.191	NA	NA	1.000	NA

*The models also include the interaction effects on the microbial assemblages. Df, degrees of freedom; SS, sum of square; MS, mean square; FR, pseudo *F*-ratio; *R*^2^, partial variance explained by the factor, Pr(>*F*), *P*-value. Colons in factors indicate interactions. The effect of habitat type was significant regardless of the permutation strategy adopted, i.e., when samples were permuted within each Population, Transect, Massif or without strata (Table A3)*.

### Fungal patterns

The variation partitioning on fungal community dissimilarities (Table 1) revealed equal and significant effects of habitat (*F*-ratio = 5.004, *R*^2^ = 0.026, *P* = 0.001), bedrock (*F*-ratio = 5.843, *R*^2^ = 0.030, *P* = 0.001), and elevation (*F*-ratio = 6.014, *R*^2^ = 0.031, *P* = 0.001) as well as a significant effect of the habitat, bedrock, and elevation interactions (*F*-ratio = 1.654, *R*^2^ = 0.009, *P* = 0. 041). The effect of habitat and elevation was significant regardless of the permutation strategy (Table A3 in Appendix). In contrast to bacteria, the fungal dissimilarities were related to SWC (*R*^2^ = 0.18, *P* = 0.001), C (*R*^2^ = 0.12, *P* = 0.001), ${\text{N - NH}}_{4}^{+}$ (*R*^2^ = 0.10, *P* = 0.001) and, to a lesser extent, ${\text{N - NO}}_{3}^{-}$ (*R*^2^ = 0.06, *P* = 0.006) and pH (*R*^2^ = 0.06, *P* = 0.007). The compositions of fungal communities in In and Out habitats were equally variable (Figure 4B, avg. distance to centroid: Out = 0.3255, In = 0.3412, *F*-value = 3.09, *P* = 0.192). However, the beta-diversity patterns in In habitat differed from that of Out habitat, as illustrated by the direction of variation for Out and In communities between siliceous and calcareous communities (Figure 4B). Community dissimilarities in In habitats increased with environmental dissimilarities, particularly C, ${\text{N - NH}}_{4}^{+}$, and SWC (Mantel test, Spearman rank ρ = 0.27, 0.25, 0.19 for C, SWC, ${\text{N - NH}}_{4}^{+}$, respectively, *P* < 0.005). The Mantel correlations were still significant when controlling for geographic distances or elevation (partial Mantel test, *P* > 0.05). Community dissimilarities in In habitat also increased with differences in elevation (Spearman rank ρ = 0.29, *P* = 0.002 and ρ = 0.22, *P* = 0.001 for calcareous and siliceous bedrock, respectively). Conversely, Out community dissimilarities were only weakly correlated with soil pH (Spearman rank ρ = 0.07, *P* < 0.001), which is significant even when controlling for geographic distance and elevation (*P* < 0.05), but did not correlate with differences in elevation (*P* > 0.05). Finally, In-Out beta-diversity was higher on siliceous bedrock and at high elevations (Figures 4B and 5B,C). However, In-Out beta-diversity did not correlate with the respective paired In-Out environmental distances (Table A2).

## Discussion

Cushion plants affect the composition of both bacterial and fungal communities weakly but significantly, despite variability in both In and Out communities (Table 1; Figure 4). This relationship was observed regardless of the strategy that was adopted in the permutation procedure that was used for variance partitioning. This result emphasizes the multiple-scale effect of cushion plants on microbial community composition, from the population level to the scale of the whole study, which spans two mountain ranges with distinct bedrock types. Interestingly, we observed contrasting responses of the bacterial and fungal communities to the biotic and abiotic environments.

The bacterial communities in Out habitat were predominantly affected by bedrock type and, to a lesser extent, elevation. By contrast, beta-diversity in In habitat was strongly reduced, indicating a convergence of the communities under the cushions on both calcareous and siliceous bedrock and at both high and low elevation. Furthermore, the In-Out bacterial beta-diversity was correlated with pH. Soil pH was thus the best and main variable for explaining the patterns of bacterial beta-diversity among In and Out habitats. We also identified a response to elevation that may be linked to the shift in the nutrient content (Table 1; Figure 3; Table A1 in Appendix) because the shift in community composition occurred on siliceous bedrock only. The strong bedrock effect on community composition led to the selection of different bacterial communities according to the bedrock (Table 1). Soil pH is a well-known key factor influencing bacterial richness and community composition (Fierer and Jackson, 2006; Hogberg et al., 2007; Zinger et al., 2011; Shahnavaz et al., 2012). On average, the soil pH varied by ∼3.5 U (from 3.5 to 8.5) among bedrock types (Figure 2) but only ∼1 U (from 6 to 8.5) among cushions that were established on calcareous and siliceous bedrock types due to an increase in the soil pH inside the cushions that were located on siliceous bedrock (Figure 2). Lauber et al. (2009) observed that the effect of pH was particularly strong in acidic conditions and, more generally, when soil pH ranged between 4 and 6, leveling off above pH 6. Our experiment was not designed to identify the source of In communities, and more information about the species distributions or dispersal would be necessary to determine their sources. However the reduction of bacterial beta-diversity, together with the similarity of certain Out and In communities that was observed in PCoA ordination (Figure 4A) and the relatively weak values of the In-Out beta-diversity (Figure 5A), suggests that bacterial communities in In habitats are likely a subset of the bacterial communities in Out habitats. Such a feature is consistent with earlier reports suggesting that the soil surrounding plants seems to constitute the main source of rhizospheric microbial communities (Berg and Smalla, 2009). By providing a more homogeneous habitat with consistently enhanced C, N, ${\text{N - NH}}_{4}^{+}$, and SWC content and buffered soil pH, the cushions of *S. acaulis* act as a biotic filter on bacterial beta-diversity that counteracts the influence of the local environmental context, particularly bedrock type, likely recruiting and/or excluding bacterial taxa that contribute to the outside cushion community variability.

The patterns of the fungal communities were less obvious and much more variable than those of the bacterial communities. The fungal communities from In habitats were as variable as those from Out habitats (Figure 4B; Table 1), with several differences. The differences between Out communities were mainly but weakly related to soil pH, reflecting differences in community compositions between bedrock types (Figure 4B). By contrast, differences between In communities correlated with soil C, ${\text{N - NH}}_{4}^{+}$, and SWC, reflecting differences between cushions established on calcareous or siliceous bedrocks and at low or high elevation (Figure 4B; Table 1). The weak effect of soil pH on fungal communities compared to that observed for bacteria has already been reported in both arable (Rousk et al., 2010) and alpine grasslands soils (Zinger et al., 2011). These studies further reveal that fungal beta-diversity is instead related to soil nutrient status, and our results confirmed this trend. The effect of pH in Out habitats is overwhelmed in In habitats by the response of fungi to nutrient content. The absence of plants in Out habitat, together with nutrient limitations and the combined disturbance of oscillating climatic extremes and soil movement typical in alpine soils (Körner, 2003), could preclude the growth and hamper the hyphal proliferation of many fungi due to their typical mycorrhizal or saprophytic status, which is supported by the difficulties we encountered in amplifying fungal DNA from these samples. Thus, we hypothesize that by providing a nutritional resource and stable substrate, cushions sustain the growth of many more fungi than do open areas. In this sense, a study in the Andean alpine ecosystem reported that cushions of *A. madreporica* contain more spores of AM fungi than retrieved on open areas (Casanova-Katny et al., 2011). Overall, there is a strong link between the soil nutritive status of cushions, the environmental context in which they established and the associated fungal communities. Furthermore, cushions counteracted the effect of pH on the fungal community composition. As observed for bacteria, fungal beta-diversity patterns in In habitats differed from the ones in Out habitats.

The higher variability observed in fungal patterns compared to bacteria and the low variability that we were able to explain could be due to several factors. We followed different molecular markers; the V3 region of the 16S rRNA gene (bacterial marker) is most likely more phylogenetically conserved than the ITS1 region (fungal marker) (Brown et al., 2005). Other studies using the same or other molecular markers have also reported that fungal community patterns are difficult to interpret, yielding minimally explanatory models (Costa et al., 2006; Mougel et al., 2006; Hovatter et al., 2011; Zinger et al., 2011), which may be due to the patchy distribution of soil fungi (Manter et al., 2010). The response of bacteria and fungi to abiotic variables was still significant after controlling for geographic distance, which supports the link between microbial community composition and its response to environmental gradients, but key environmental variables or the appropriate spatial scale may also not have been considered for fungi.

Finally, the magnitude of the change in the microbial community composition between In and Out habitats (In-Out beta-diversity) varied along environmental gradients (Figure 5). The In-Out bacterial beta-diversity was correlated with the modification of soil pH and was, therefore, higher on siliceous bedrock (Figure 5A; Table A2 in Appendix). Moreover, bacterial communities responded to elevation solely in Out habitat on siliceous bedrock, supporting a strong cushion effect on siliceous bedrock. Because the fungi responded more strongly to soil nutrients, the In-Out fungal beta-diversity was higher on siliceous bedrock and at high elevations (Figure 5B; Table A2 in Appendix). On siliceous bedrock and at high elevations, conditions in Out habitat were more acidic (siliceous bedrock) and nutrient-limited (siliceous bedrock and at high elevation). Interestingly, the cushions located in these constrained conditions were richer in nutrients than cushions located in less constrained conditions, and strongly modified the soil pH (Figures 2 and 3), resulting in significant and even stronger ecosystem engineering. Although the In-Out fungal beta-diversity did not correlate with the respective abiotic modifications, it was generally higher at sites where the abiotic modification was higher. The fact that we did not observe pair-wise relationships may be because different factors structured the fungal communities in In and Out habitats. Thus, our observations indicate that the link between microbes and abiotic modification depends on the intensity of the abiotic stress mitigated by engineering organisms, as concluded in earlier works (Wright et al., 2006; Navel et al., 2012), but we suggest that a plant factor could be involved, either directly (via intraspecific variability) or indirectly (via physiological response to environmental harshness). Overall, these results suggest that the selective effect of cushions on bacterial and fungal communities could be particularly strong under more extreme conditions.

There is a growing body of evidence suggesting that cushion plants represent benefactor species or “nurse plants” that facilitate the recruitment of other plants (Arroyo et al., 2003; Cavieres et al., 2006; Antonsson et al., 2009) and arthropods (Molina-Montenegro et al., 2006; Molenda et al., 2012). Several studies have reported that the magnitude of facilitation by cushion plants, including *S. acaulis*, increases with environmental stress in alpine and arctic biomes on several mountains worldwide (Arroyo et al., 2003; Cavieres et al., 2006; Antonsson et al., 2009; Yang et al., 2010; Anthelme et al., 2012). These studies note that the local amelioration of climatic variables (e.g., temperatures) is involved (Arroyo et al., 2003; Cavieres et al., 2007), although nutrient enrichment is also a factor (Yang et al., 2010; Anthelme et al., 2012) and is more pronounced under stressful conditions, as observed here for nutrients, water and microbial communities. Our study opens a new avenue to understanding the ecology of these nurse plants from a microbial perspective. Several studies have reported that microbes act as the third party in plant–plant interactions (Callaway et al., 2007). Further work is needed to establish the linkages between plant–microbe interactions and plant–plant interactions in this particular model. For instance, it has been demonstrated that native plant species growing inside cushions in Andean alpine environments display enhanced AM mycorrhizal status compared to those growing outside (Casanova-Katny et al., 2011). Different functional groups of microbes can have different roles in the functioning of the cushion system or different links with the plant species that grow inside cushions. In this study, we demonstrated that bacteria and fungi differed in their relationships to the modification of soil abiotic properties by cushions. Additional studies are needed to disentangle the influence of microbial communities on the recruitment of taxa at higher trophic levels as well as how that recruitment influences the microbial communities that are associated with cushions. Plants growing inside cushions could influence the composition of soil microbes and their response to environmental gradients through mycorrhizal interactions or by providing new sources of organic matter.

It is possible that the cushion effect that was observed here could be applicable to other non-cushion-forming alpine plants because of possible similar abiotic modifications via organic matter deposition. Nevertheless, our results support the view that *S. acaulis* is a foundation species in the alpine ecosystem (Molenda et al., 2012) because of its effect on the structure of many trophic levels (Antonsson et al., 2009; Molenda et al., 2012). Overall, cushions constitute a unique habitat in extreme cliff ecosystems with modified local habitat conditions. Their presence significantly affects the beta-diversity patterns of bacterial and fungal communities. The bacterial and fungal communities do not respond similarly to the presence of cushions, likely because they are not sensitive to the same set of abiotic soil parameters. For both communities, the plants induce different responses to bedrock and elevation compared to the outside, but the response was inverse between bacteria and fungi; while plants had a buffering effect on bacterial communities, they exacerbated the response of fungi to bedrock and elevation. Our results support that habitat type differentially influences the distribution of soil microbes (Fierer et al., 2011; Hovatter et al., 2011) and that their response to environmental gradients depends on the taxa and microbial domain investigated (Singh et al., 2012; Wang et al., 2012). We demonstrated that there might be fundamental differences in the mechanisms underlying these molecular diversity patterns. Dissimilarities in the bacterial and fungal community between In and Out habitats were both positively related to the magnitude of habitat modification (i.e., higher in harsher conditions). Our results stress the need for integrated studies of the nurse effect of alpine cushions in which great attention should be paid to the role of microorganisms and their distribution in determining the outcome of plant–plant interactions and the biogeochemical functioning of these islands of fertility.

## Conflict of Interest Statement

The authors declare that the research was conducted in the absence of any commercial or financial relationships that could be construed as a potential conflict of interest.

## Appendix

**Table A1:** **Model selection of fixed effects and interactions explaining the variation in soil properties**.

Soil characteristic	Model	*p*	AIC	deltaAIC
Soil C content	Habitat + elevation + bedrock + habitat: bedrock + elevation: habitat	6	1102.6	0
	Habitat + elevation + bedrock + elevation: bedrock + elevation: habitat + habitat: bedrock	7	1103.8	1.2
	Habitat + bedrock + habitat: bedrock	4	1104.3	1.7
	Habitat × elevation × bedrock	8	1105.7	3.1
	Habitat + elevation + bedrock + habitat: bedrock	5	1106.1	3.5
	Habitat + elevation + bedrock + habitat: bedrock + elevation: bedrock	6	1107.3	4.7
	Habitat + elevation + elevation: habitat	4	1126.1	23.5
	Habitat + elevation + bedrock + habitat: elevation	5	1126.7	24.1
	Habitat + elevation + bedrock + elevation: bedrock + elevation: habitat	6	1127.7	25.1
	Habitat	2	1139	36.4
	Habitat + bedrock	3	1140.1	37.5
	Habitat + elevation	3	1140.9	38.3
	Habitat + elevation + bedrock	4	1141.8	39.2
	Habitat + elevation + bedrock + bedrock: elevation	5	1142.8	40.2
	1	1	1220.3	117.7
	Bedrock	2	1221.8	119.2
	Elevation	2	1222.3	119.7
	Bedrock + elevation	3	1223.8	121.2
	Elevation + bedrock + elevation: bedrock	4	1225.3	122.7
Soil N content	Habitat + elevation + bedrock + habitat: bedrock + elevation: habitat	6	82.3	0
	Habitat + elevation + bedrock + habitat: bedrock	5	83.2	0.9
	Habitat + elevation + bedrock + elevation: bedrock + elevation: habitat + habitat: bedrock	7	83.9	1.6
	Habitat + elevation + bedrock + habitat: bedrock + elevation: bedrock	6	84.7	2.4
	Habitat + elevation + elevation: habitat	4	85.3	3
	Habitat × elevation × bedrock	8	85.9	3.6
	Habitat + elevation + bedrock + habitat: elevation	5	86.6	4.3
	Habitat + elevation + bedrock + elevation: bedrock + elevation: habitat	6	88.2	5.9
	Habitat + bedrock + habitat: bedrock	4	90.2	7.9
	Habitat + elevation	3	90.6	8.3
	Habitat + elevation + bedrock	4	92	9.7
	Habitat + elevation + bedrock + bedrock: elevation	5	93.5	11.2
	Habitat	2	95.7	13.4
	Habitat + bedrock	3	97.7	15.4
	Elevation	2	130.4	48.1
	Bedrock + elevation	3	131.6	49.3
	Elevation + bedrock + elevation: bedrock	4	133.4	51.1
	1	1	133.6	51.3
	Bedrock	2	135.6	53.3
Soil N-NO3 content	Habitat × elevation × bedrock	8	1070.2	0
	Elevation + bedrock + elevation: bedrock	4	1071	0.8
	Elevation	2	1071.6	1.4
	Bedrock + elevation	3	1072	1.8
	Habitat + elevation + bedrock + bedrock: elevation	5	1072.5	2.3
	Habitat + elevation	3	1073.2	3
	Habitat + elevation + bedrock	4	1073.6	3.4
	Habitat + elevation + bedrock + habitat: bedrock + elevation: bedrock	6	1074.1	3.9
	Habitat + elevation + bedrock + elevation: bedrock + elevation: habitat	6	1074.2	4
	Habitat + elevation + elevation: habitat	4	1074.8	4.6
	Habitat + elevation + bedrock + habitat: bedrock	5	1075.1	4.9
	Habitat + elevation + bedrock + habitat: elevation	5	1075.2	5
	Habitat + elevation + bedrock + elevation: bedrock + elevation: habitat + habitat: bedrock	7	1075.2	5
	Habitat + elevation + bedrock + habitat: bedrock + elevation: habitat	6	1076.3	6.1
	Bedrock	2	1078.2	8
	1	1	1078.8	8.6
	Habitat + bedrock	3	1079.8	9.6
	Habitat	2	1080.3	10.1
	Habitat + bedrock + habitat: bedrock	4	1081.4	11.2
Soil N-NH4 content	Habitat + elevation + bedrock + habitat: bedrock + elevation: habitat	6	1476.3	0
	Habitat + bedrock + habitat: bedrock	4	1476.9	0.6
	Habitat + elevation + bedrock + elevation: bedrock + elevation: habitat + habitat: bedrock	7	1477.3	1
	Habitat + elevation + bedrock + habitat: bedrock	5	1478.1	1.8
	Habitat × elevation × bedrock	8	1478.1	1.8
	Habitat + elevation + bedrock + habitat: bedrock + elevation: bedrock	6	1479.1	2.8
	Habitat + elevation + elevation: habitat	4	1482.6	6.3
	Habitat + elevation + bedrock + habitat: elevation	5	1482.8	6.5
	Habitat + elevation + bedrock + elevation: bedrock + elevation: habitat	6	1483.7	7.4
	Habitat + bedrock	3	1489.5	13.2
	Habitat	2	1489.6	13.3
	Habitat + elevation + bedrock	4	1490.6	14.3
	Habitat + elevation	3	1490.7	14.4
	Habitat + elevation + bedrock + bedrock: elevation	5	1491.5	15.2
	Bedrock	2	1547.6	71.3
	1	1	1548.7	72.4
	Bedrock + elevation	3	1548.8	72.5
	Elevation	2	1549.9	73.6
	Elevation + bedrock + elevation: bedrock	4	1550.1	73.8
Soil water content	Habitat + elevation + bedrock + habitat: bedrock + elevation: habitat	6	239.8	0
	Habitat + elevation + bedrock + elevation: bedrock + elevation: habitat + habitat: bedrock	7	241.5	1.7
	Habitat × elevation × bedrock	8	241.8	2
	Habitat + elevation + elevation: habitat	4	242.8	3
	Habitat + elevation + bedrock + habitat: elevation	5	244.7	4.9
	Habitat + elevation + bedrock + elevation: bedrock + elevation: habitat	6	246.5	6.7
	Habitat + elevation + bedrock + habitat: bedrock	5	254.9	15.1
	Habitat + elevation + bedrock + habitat: bedrock + elevation: bedrock	6	256.6	16.8
	Habitat + bedrock + habitat: bedrock	4	257.3	17.5
	Habitat + elevation	3	268.4	28.6
	Habitat + elevation + bedrock	4	270.3	30.5
	Habitat	2	270.9	31.1
	Habitat + elevation + bedrock + bedrock: elevation	5	272.1	32.3
	Habitat + bedrock	3	272.9	33.1
	Elevation	2	330.9	91.1
	1	1	332.9	93.1
	Bedrock + elevation	3	332.9	93.1
	Elevation + bedrock + elevation: bedrock	4	334.5	94.7
	Bedrock	2	334.7	94.9
Soil pH	Habitat + bedrock + habitat: bedrock	4	368.8	0
	Habitat + elevation + bedrock + habitat: bedrock	5	370.4	1.6
	Habitat + elevation + bedrock + habitat: bedrock + elevation: bedrock	6	370.5	1.7
	Habitat + elevation + bedrock + habitat: bedrock + elevation: habitat	6	372.4	3.6
	Habitat + elevation + bedrock + elevation: bedrock + elevation: habitat + habitat: bedrock	7	372.5	3.7
	Habitat × elevation × bedrock	8	374.4	5.6
	Habitat + elevation + bedrock + elevation: bedrock + elevation: habitat	6	400.4	31.6
	Habitat + elevation + bedrock + habitat: elevation	5	400.6	31.8
	Habitat + bedrock	3	401.1	32.3
	Habitat + elevation + bedrock + bedrock: elevation	5	402.7	33.9
	Habitat + elevation + bedrock	4	402.9	34.1
	Habitat + elevation + elevation: habitat	4	417.8	49
	Habitat + elevation	3	419.9	51.1
	Habitat	2	420.7	51.9
	Bedrock	2	458.4	89.6
	Elevation + bedrock + elevation: bedrock	4	460.3	91.5
	Bedrock + elevation	3	460.6	91.8
	Elevation	2	476.7	107.9
	1	1	477.7	108.9

*Fixed effects are the habitat (In-Out), bedrock, and elevation. Models are ordered according to the AIC statistic. *p* is the number of parameters in the model*.

**Table A2:** **Model selection of fixed effects and interactions explaining the variation in bacterial and fungal In-Out beta-diversity**.

In-Out beta-diversity	Model	*p*	AIC	deltaAIC
Bacteria	Dist.pH	2	−488.7	0
	Dist.env	2	−486.9	1.8
	Bedrock + alt	3	−483.9	4.8
	Bedrock	2	−482.9	5.8
	Alt + bedrock + alt: bedrock	4	−482.3	6.3
	Alt × bedrock	4	−482.3	6.3
	Dist.NO_3_	2	−480.9	7.8
	Dist.C	2	−475.5	13.2
	Alt	2	−475.2	13.5
	Dist.N	2	−474.7	14
	1	1	−471.8	16.9
	Dist.NH_4_	2	−470.3	18.4
	Dist.SWC	2	−469.9	18.8
Fungi	Bedrock + alt	3	−267.1	0
	Bedrock	2	−265.8	1.3
	Alt + bedrock + alt: bedrock	4	−265.1	2
	Alt × bedrock	4	−265.1	2
	Dist.NO_3_	2	−258.4	8.7
	1	1	−258.4	8.7
	Dist.pH	2	−258.1	9
	Alt	2	−258	9.1
	Dist.C	2	−257.7	9.4
	Dist.env	2	−257.3	9.8
	Dist.N	2	−256.9	10.2
	Dist.SWC	2	−256.7	10.4
	Dist.NH_4_	2	−256.4	10.7

*Fixed effects are the bedrock, elevation, and the dissimilarities of total carbon content, total nitrogen content, soil water content, ammonium content, nitrate content, and soil pH. Models are ordered according to the AIC statistic. *p* is the number of parameters in the model*.

**Table A3:** **Results of the different permutation strategies used in the non-parametric multivariate analysis of variance**.

		Pr(>*F*) no strata	Pr(>*F*) strata pop	Pr(>*F*) strata transect	Pr(>*F*) strata massifs
Bacteria	Habitat	0.001	0.001	0.001	0.001
	Bedrock	_	_	_	_
	Elevation	0.004	_	0.002	0.005
	Habitat: bedrock	0.001	_	0.001	0.001
	Habitat: elevation	0.666	_	0.720	0.686
	Bedrock: elevation	0.070	_	0.058	0.092
	Habitat: bedrock: elevation	0.126	_	0.122	0.131
	Residuals	NA	NA	NA	NA
	Total	NA	NA	NA	NA
Fungi	Habitat	0.001	0.001	0.001	0.001
	Bedrock	_	_	_	_
	Elevation	0.001	_	0.001	0.001
	Habitat: bedrock	0.002	_	0.003	0.002
	Habitat: elevation	0.199	_	0.192	0.182
	Bedrock: elevation	0.010	_	0.007	0.009
	Habitat: bedrock: elevation	0.041	_	0.043	0.032
	Residuals	NA	NA	NA	NA
	Total	NA	NA	NA	NA

*The analysis is based on Bray–Curtis dissimilarity matrices of microbial SSCP profiles. “_” indicates meaningless permutation procedure. The samples permutation procedure did not influence the significance of the effects*.

## References

[B1] Akaike H. (1974). New look at the statistical model identification. IEEE Trans. Automat. Contr. 19, 716–723 10.1109/TAC.1974.1100707

[B2] Anderson M. J. (2001). A new method for non-parametric multivariate analysis of variance. Austral Ecol. 26, 32–46 10.1111/j.1442-9993.2001.01070.pp.x

[B3] Anderson M. J. Ellingsen K. E. McArdle B. H. (2006). Multivariate dispersion as a measure of beta-diversity. Ecol. Lett. 9, 683–693 10.1111/j.1461-0248.2006.00926.x 16706913

[B4] Anthelme F. Buendia B. Mazoyer C. Dangles O. (2012). Unexpected mechanisms sustain the stress gradient hypothesis in a tropical alpine environment. J. Veg. Sci. 23, 62–72 10.1111/j.1654-1103.2011.01333.x

[B5] Antonsson H. Bjork R. G. Molau U. (2009). Nurse plant effect of the cushion plant *Silene acaulis* (L.) Jacq. in an alpine environment in the subarctic Scandes, Sweden. Plant Ecol. Divers (Print) 2, 17–25 10.1080/17550870902926504

[B6] Arroyo M. T. K. Cavieres L. A. Penaloza A. Arroyo-Kalin M. A. (2003). Positive associations between the cushion plant *Azorella monantha* (Apiaceae) and alpine plant species in the Chilean Patagonian Andes. Plant Ecol. 169, 121–129 10.1023/A:1026281405115

[B7] Badano E. I. Cavieres L. A. (2006). Impacts of ecosystem engineers on community attributes: effects of cushion plants at different elevations of the Chilean Andes. Divers. Distrib. 12, 388–396 10.1111/j.1366-9516.2006.00248.x

[B8] Badano E. I. Molina-Montenegro M. A. Quiroz C. Cavieres L. A. (2002). Effects of the cushion plant *Oreopolus glacialis* (Rubiaceae) on species richness and diversity in a high-Andean plant community of central Chile. Rev. Chil. Hist. Nat. 75, 757–765 10.4067/S0716-078X2002000400011

[B9] Bais H. P. Weir T. L. Perry L. G. Gilroy S. Vivanco J. M. (2006). The role of root exudates in rhizosphere interactions with plants and other organisms. Annu. Rev. Plant Biol. 57, 233–266 10.1146/annurev.arplant.57.032905.105159 16669762

[B10] Bates D. M. (2010). lme4: Mixed-Effects Modeling with R. New York: Springer

[B11] Bates D. Maechler M. Bolker B. (2011). lme4: Linear Mixed-Effects Models Using S4 Classes. R Package Version 0.999375-39. Available at: http://CRAN.R-project.org/package=lme4

[B12] Berg G. Smalla K. (2009). Plant species and soil type cooperatively shape the structure and function of microbial communities in the rhizosphere. FEMS Microbiol. Ecol. 68, 1–13 10.1111/j.1574-6941.2009.00654.x 19243436

[B13] Brown M. V. Schwalbach M. S. Hewson I. Fuhrman J. A. (2005). Coupling 16S-ITS rDNA clone libraries and automated ribosomal intergenic spacer analysis to show marine microbial diversity: development and application to a time series. Environ. Microbiol. 7, 1466–1479 10.1111/j.1462-2920.2005.00835.x 16104869

[B14] Bryant J. A. Lamanna C. Morlon H. Kerkhoff A. J. Enquist B. J. Green J. L. (2008). Microbes on mountainsides: contrasting elevational patterns of bacterial and plant diversity. Proc. Natl. Acad. Sci. U.S.A. 105, 11505–11511 10.1073/pnas.0801920105 18695215 PMC2556412

[B15] Callaway R. M. Howard T. G. Esser K. Löttge U. Beyschlag W. Murata J. (2007). Competitive Networks, Indirect Interactions, and Allelopathy: A Microbial Viewpoint on Plant Communities Progress in Botany. Berlin: Springer

[B16] Casanova-Katny M. A. Torres-Mellado G. A. Palfner G. Cavieres L. A. (2011). The best for the guest: high Andean nurse cushions of *Azorella madreporica* enhance arbuscular mycorrhizal status in associated plant species. Mycorrhiza 21, 613–622 10.1007/s00572-011-0367-1 21384201

[B17] Cavieres L. A. Badano E. I. Sierra-Almeida A. Gomez-Gonzalez S. Molina-Montenegro M. A. (2006). Positive interactions between alpine plant species and the nurse cushion plant *Laretia acaulis* do not increase with elevation in the Andes of central Chile. New Phytol. 169, 59–69 10.1111/j.1469-8137.2005.01573.x 16390419

[B18] Cavieres L. A. Badano E. I. Sierra-Almeida A. Molina-Montenegro M. A. (2007). Microclimatic modifications of cushion plants and their consequences for seedling survival of native and non-native herbaceous species in the high Andes of central Chile. Arct. Antarct. Alp. Res. 39, 229–236 10.1657/1523-0430(2007)39[229:MMOCPA]2.0.CO;2

[B19] Costa R. Gotz M. Mrotzek N. Lottmann J. Berg G. Smalla K. (2006). Effects of site and plant species on rhizosphere community structure as revealed by molecular analysis of microbial guilds. FEMS Microbiol. Ecol. 56, 236–249 10.1111/j.1574-6941.2005.00026.x 16629753

[B20] Delbes C. Moletta R. Godon J. (2000). Monitoring of activity dynamics of an anaerobic digester bacterial community using 16S rRNA polymerase chain reaction – single-strand conformation polymorphism analysis. Environ. Microbiol. 2, 506–515 10.1046/j.1462-2920.2000.00132.x 11233159

[B21] Eskelinen A. Stark S. Mannisto M. (2009). Links between plant community composition, soil organic matter quality and microbial communities in contrasting tundra habitats. Oecologia 161, 113–123 10.1007/s00442-009-1362-5 19452173

[B22] Eviner V. T. Chapin F. S. (2003). Functional matrix: a conceptual framework for predicting multiple plant effects on ecosystem processes. Annu. Rev. Ecol. Evol. Syst. 34, 455–485 10.1146/annurev.ecolsys.34.011802.132342

[B23] Fierer N. Jackson R. B. (2006). The diversity and biogeography of soil bacterial communities. Proc. Natl. Acad. Sci. U.S.A. 103, 626–631 10.1073/pnas.0507535103 16407148 PMC1334650

[B24] Fierer N. McCain C. M. Meir P. Zimmermann M. Rapp J. M. Silman M. R. (2011). Microbes do not follow the elevational diversity patterns of plants and animals. Ecology 92, 797–804 10.1890/10-1170.1 21661542

[B25] Hartmann A. Schmid M. Van Tuinen D. Berg G. (2009). Plant-driven selection of microbes. Plant Soil 321, 235–257 10.1007/s11104-008-9814-y

[B26] Hogberg M. N. Hogberg P. Myrold D. D. (2007). Is microbial community composition in boreal forest soils determined by pH, C-to-N ratio, the trees, or all three? Oecologia 150, 590–601 10.1007/s00442-006-0562-5 17033802

[B27] Hovatter S. R. Dejelo C. Case A. L. Blackwood C. B. (2011). Metacommunity organization of soil microorganisms depends on habitat defined by presence of *Lobelia siphilitica* plants. Ecology 92, 57–65 10.1890/10-0332.1 21560676

[B28] Jones C. G. Lawton J. H. Shachak M. (1994). Organisms as ecosystem engineers. Oikos 69, 373–386 10.2307/3545850

[B29] Jones C. G. Lawton J. H. Shachak M. (1997). Positive and negative effects of organisms as physical ecosystem engineers. Ecology 78, 1946–1957 10.1890/0012-9658(1997)078[2569:CDIADC]2.0.CO;2

[B30] Körner C. (2003). Alpine Plant Life. Functional Plant Ecology of High Mountain Ecosystems. Berlin: Springer

[B31] Kowalchuk G. A. Buma D. S. De Boer W. Klinkhamer P. G. L. Van Veen J. A. (2002). Effects of above-ground plant species composition and diversity on the diversity of soil-borne microorganisms. Antonie Van Leeuwenhoek 81, 509–520 10.1023/A:1020565523615 12448746

[B32] Lauber C. L. Hamady M. Knight R. Fierer N. (2009). Pyrosequencing-based assessment of soil pH as a predictor of soil bacterial community structure at the continental scale. Appl. Environ. Microbiol. 75, 5111–5120 10.1128/AEM.00335-09 19502440 PMC2725504

[B33] Lauber C. L. Strickland M. S. Bradford M. A. Fierer N. (2008). The influence of soil properties on the structure of bacterial and fungal communities across land-use types. Soil Biol. Biochem. 40, 2407–2415 10.1016/j.soilbio.2008.05.021

[B34] Legendre P. Legendre L. (1998). Numerical Ecology. Amsterdam: Elsevier Science

[B35] Manter D. K. Weir T. L. Vivanco J. M. (2010). Negative effects of sample pooling on PCR-based estimates of soil microbial richness and community structure. Appl. Environ. Microbiol. 76, 2086–2090 10.1128/AEM.03017-09 20139317 PMC2849261

[B36] Molenda O. Reid A. Lortie C. J. (2012). The alpine cushion plant *Silene acaulis* as foundation species: a bug’s-eye view to facilitation and microclimate. PLoS ONE 7:e37223 10.1371/journal.pone.0037223 22655035 PMC3360034

[B37] Molina-Montenegro M. A. Badano E. I. Cavieres L. A. (2006). Cushion plants as microclimatic shelters for two ladybird beetles species in alpine zone of central Chile. Arct. Antarct. Alp. Res. 38, 224–227 10.1657/1523-0430(2006)38[224:CPAMSF]2.0.CO;2

[B38] Mougel C. Offre P. Ranjard L. Corberand T. Gamalero E. Robin C. (2006). Dynamic of the genetic structure of bacterial and fungal communities at different developmental stages of *Medicago truncatula* Gaertn. cv. Jemalong line J5. New Phytol. 170, 165–175 10.1111/j.1469-8137.2006.01650.x 16539613

[B39] Navel S. Mermillod-Blondin F. Montuelle B. Chauvet E. Marmonier P. (2012). Sedimentary context controls the influence of ecosystem engineering by bioturbators on microbial processes in river sediments. Oikos 121, 1134–1144 10.1111/j.1600-0706.2011.19742.x

[B40] Oksanen J. Guillaume Blanchet F. Kindt R. Legendre P. O’Hara R. B. Simpson G. L. (2011). vegan: Community Ecology Package. R Package Version 1.17-6. Available at: http://CRAN.R-project.org/package=vegan

[B41] Pinheiro J. C. Bates D. M. (2000). Mixed-Effects Models in S and S-Plus. New York: The Statistics and Computing Series, Springer-Verlag

[B42] R Development Core Team (2011). “R: A Language and Environment for Statistical Computing”. Vienna: R Foundation for Statistical Computing Available at: http://www.R-project.org.

[B43] Robertson G. P. Coleman D. C. Bledsoe C. S. Sollins P. (1999). Standard Soil Methods for Long-Term Ecological Research. New York: Oxford University Press

[B44] Rousk J. Baath E. Brookes P. C. Lauber C. L. Lozupone C. Caporaso J. G. (2010). Soil bacterial and fungal communities across a pH gradient in an arable soil. ISME J. 4, 1340–1351 10.1038/ismej.2010.58 20445636

[B45] Shahnavaz B. Zinger L. Lavergne S. Choler P. Geremia R. A. (2012). Phylogenetic clustering reveals selective events driving the turnover of bacterial community in alpine tundra soils. Arct. Antarct. Alp. Res. 44, 232–238 10.1657/1938-4246-44.2.232

[B46] Singh D. Takahashi K. Kim M. Chun J. Adams J. M. (2012). A hump-backed trend in bacterial diversity with elevation on Mount Fuji, Japan. Microb. Ecol. 63, 429–437 10.1007/s00248-011-9900-1 21735154

[B47] Sklenar P. (2009). Presence of cushion plants increases community diversity in the high equatorial Andes. Flora 204, 270–277 10.1016/j.flora.2008.04.001

[B48] van der Heijden M. G. A. Bardgett R. D. Van Straalen N. M. (2008). The unseen majority: soil microbes as drivers of plant diversity and productivity in terrestrial ecosystems. Ecol. Lett. 11, 296–310 10.1111/j.1461-0248.2007.01139.x 18047587

[B49] Wang J. J. Soininen J. Zhang Y. Wang B. X. Yang X. D. Shen J. (2012). Patterns of elevational beta-diversity in micro- and macro-organisms. Glob. Ecol. Biogeogr. 21, 743–750 10.1111/j.1466-8238.2011.00718.x

[B50] White T. Bruns T. Lee S. Taylor J. (1990). PCR Protocols: A Guide to Methods and Applications Amplification and Direct Sequencing of Fungal Ribosomal RNA Genes for Phylogenetics. San Diego: Academic Press

[B51] Wright J. P. Jones C. G. Boeken B. Shachak M. (2006). Predictability of ecosystem engineering effects on species richness across environmental variability and spatial scales. J. Ecol. 94, 815–824 10.1111/j.1365-2745.2006.01132.x

[B52] Yang Y. Niu Y. Cavieres L. A. Sun H. (2010). Positive associations between the cushion plant *Arenaria polytrichoides* (Caryophyllaceae) and other alpine plant species increase with altitude in the Sino-Himalayas. J. Veg. Sci. 21, 1048–1057 10.1111/j.1654-1103.2010.01215.x

[B53] Zinger L. Gury J. Alibeu O. Rioux D. Gielly L. Sage L. (2008). CE-SSCP and CE-FLA, simple and high-throughput alternatives for fungal diversity studies. J. Microbiol. Methods 73, 209 10.1016/j.mimet.2008.03.001 18054096

[B54] Zinger L. Gury J. Giraud F. Krivobok S. Gielly L. Taberlet P. (2007). Improvements of polymerase chain reaction and capillary electrophoresis single-strand conformation polymorphism methods in microbial ecology: toward a high-throughput method for microbial diversity studies in soil. Microb. Ecol. 54, 203–216 10.1007/s00248-006-9151-8 17587075

[B55] Zinger L. Lejon D. P. H. Baptist F. Bouasria A. Aubert S. Geremia R. A. (2011). Contrasting diversity patterns of crenarchaeal, bacterial and fungal soil communities in an alpine landscape. PLoS ONE 6:e19950 10.1371/journal.pone.0019950 21589876 PMC3093402

